# Chemical Composition and Nutritional Value of Royal Jelly Samples Obtained from Honey Bee (*Apis mellifera*) Hives Fed on Oak and Rapeseed Pollen Patties

**DOI:** 10.3390/insects15030141

**Published:** 2024-02-21

**Authors:** Sampat Ghosh, Chuleui Jung

**Affiliations:** 1 Agriculture Science and Technology Research Institute, Andong National University, Andong 36729, Gyeonbuk, Republic of Korea; sampatghosh.bee@gmail.com; 2 Department of Plant Medicals, Andong National University, Andong 36729, Gyeongbuk, Republic of Korea

**Keywords:** honey bee, *Apis mellifera*, bee pollen, pollen patty, protein, amino acid, zinc, iron, nutrition supplement

## Abstract

**Simple Summary:**

We investigated whether honey bee colonies adjust the nutritional quality of royal jelly based on the pollen patties they are fed. Pollen patties made of oak or rapeseed bee pollen were given to young worker bees, and the harvested royal jelly was analyzed for its chemical composition, which is of nutritional importance. Surprisingly, the nutritional levels of the pollen patties did not significantly impact the overall composition of the royal jelly, except for the crude fat. Despite differences in the protein intensity, 10-HDA content, the key indicator of the royal jelly quality, remained consistent between the oak and rapeseed pollen patty treatments. The findings suggest that honey bees possess a mechanism to compensate for nutritional variations in pollen patties.

**Abstract:**

Young workers, i.e., nurse honey bees, synthesize and secrete royal jelly to feed the brood and queen. Since royal jelly is a protein-rich substance, the quality of royal jelly may be influenced by the consumption of feed with varying protein content. We tested whether honey bee (*Apis mellifera*) colonies compensates for the nutritional quality to produce royal jelly by feeding different pollen patties made of oak or rapeseed pollen. After harvesting royal jelly, we examined the chemical composition including proximate nutrients, amino acids, proteins, fatty acids, and minerals of royal jelly samples obtained from two treatments. The results revealed that pollen patties with different nutritional levels did not influence the nutritional composition except for the crude fat. The levels of 10-HDA, which serves as an indicator of the royal jelly quality, showed no significant difference between the oak and rapeseed treatments, with values of 1.9 and 2.1 g/100 g, respectively. However, we found some differences in the protein intensity, particularly the MRJP3 precursor, MRJP3-like, and glucose oxidase. This study suggests that honey bees may have mechanisms to compensate for nutritional standards to meet the brood’s and queen’s nutritional requirements during bee pollen collection, preserving bee bread and royal jelly secretion.

## 1. Introduction

Royal jelly (RJ) is a substance secreted from the hypopharyngeal and mandibular glands of young worker (5–15 days old nurse) honey bees [1]. It plays a crucial role in the caste determination of honey bees [2]. A fertile queen is developed by feeding on RJ. The nutritional source affects the gene silencing of DNA methyltransferase Dnmt 3, leading to the emergence of a fertile queen honey bee that shares genetic identity with sterile adult worker honey bees [3]. Thus, in order to understand the component that triggers its function, the study of the chemical composition of RJ is of the utmost importance. Chemical analyses reveal that it contains water (60–70%), protein (12–18%), carbohydrate (10–16%), lipid (3–6%), minerals, vitamins (thiamin, riboflavin, pantothenic acid, pyridoxine, niacin, folic acid, inositol, and biotin), and phenolic content [4,5,6,7]. About 185 organic compounds are present in RJ, among which royalactin is the predominating protein. Among others, nucleotides (i.e., uridine, guanosine, adenosine, cytosine), acetylcholine, and gluconic acid were remarkably present in RJ [8,9,10,11]. 10-hydroxy-2-decenoic acid (10-HDA), found in substantial quantities in royal jelly (RJ), demonstrates immunomodulatory, anti-microbial, and anti-tumor properties [12,13]. Additionally, it contributes to reproductive health by promoting the synthesis of ovulation hormones and reducing the expression of follicle-stimulating hormone (FSH) and luteinizing hormone (LH) in young ovarian cells [14,15,16]. Approximately 15% of RJ is made up of proteins, specifically major royal jelly proteins (MRJPs), with nine of them identified as the most abundant, known as MRJP1–9. MRJP1–3 and MRJP5 collectively make up 82–90% of the total proteins in RJ [17,18]. The levels of expression and protein content of MRJP1–5 and MRJP7 exhibit an age-dependent pattern in the hypopharyngeal glands and brains of worker bees, with the brains showing a relatively lower abundance compared to the glands. The expression increases post hatching until the nurse bee phase, followed by a decline in older workers engaged in foraging [18]. In contrast, MRJP6 expression differs significantly from other MRJPs, remaining relatively stable in the brain but displaying peak expression in the hypopharyngeal glands during the forager period. Notably, MRJP6 is unique as its transcript abundance does not correlate with protein levels. Additionally, MRJP8 and MRJP9 demonstrate notably low expression in both tissues compared to other MRJPs [18].

Given its exceptional biological properties, RJ is receiving considerable attention and commercial appeal from industries including pharmaceutical, functional food, as well as cosmetics and manufacturing industry [7]. Concern has been reflected in attempt to standardize the quality of RJ by several countries such as Switzerland, Bulgaria, Brazil, and Uruguay [7,19]. Countries like China, Japan, the USA, Canada, Australia, and some European countries stand at the forefront of RJ production. So far, in Korea, RJ stands as the third most significant beekeeping product in the industry, valued at ￦28.1 billion [20]. Despite the absence of official production statistics, the current domestic output of RJ in Korea falls short of meeting the domestic demand [21]. Consequently, a substantial portion of the supply relies on imports. According to the latest data from the Korea Trade Statistics Service (TRASS), RJ imports have consistently risen from 27,726 kg in 2016 to 79,920 kg in 2020 [21,22].

Various factors can impact the production of RJ, including genetics, the internal population condition of colonies, food supply, and external environmental factors influenced by weather conditions. A study by Ibrahim indicates that queenless colonies tend to produce a greater quantity of RJ compared to queenright colonies [23]. Bee race itself is identified as another influencing factor on RJ production [24]. In order to understand the RJ production of honey bees, it is important to focus few parameters on which the production of jelly or jelly proteins depends. These regulatory factors include pollen quality and quantity resulting in bee bread storage and consumption, and its related worker physiology reflected by vitellogenin and juvenile hormone titers. Pollen consumption is positively correlated with the development of the hypopharyngeal gland [25,26], which is specialized for jelly production. Also, the consumption of pollen increases vitellogenin expression and vitellogenin levels. Worker honey bees fed on a 50% pollen diet had higher vitellogenin levels than workers fed on a 15% pollen diet [27]. Vitellogenin, massively present in the haemolymph of worker honey bees during the period when these nurse bees feed growing larvae with jelly [28,29], is a probable source for the proteinaceous RJ synthesis [30]. Nonetheless, the impact of consuming pollen from various floral sources on the quality of royal jelly remains largely unexplored. Typically, beekeepers supply hives with pollen patties composed of diverse bee pollens, especially in early spring, to fortify the colony. These pollen patties consist of various bee pollens, and changes in composition occur during their processing, primarily influenced by the bee pollen utilized. Notably, the nutrient content of oak and rapeseed bee pollen differs; for instance, the protein content of rapeseed pollen (26.8%) surpasses that of oak pollen (23.2%) [31,32]. Consequently, the nutrient composition of pollen patties created with these bee pollens may vary. Therefore, our study aimed to investigate the nutrient composition of royal jelly produced by honey bee colonies that were fed on pollen patties from two distinct floral sources: oak pollen patty and rapeseed pollen patty.

## 2. Materials and Methods

### 2.1. Materials

This study was carried out in collaboration with a local apiary situated close to the Andong National University. Ten healthy honey bee colonies were recruited for the study. Within the apiary, we allocated two different treatments for two months. Two different types of RJ were based on the feed provided to the honey bee colonies. Five bee colonies (*n* = 5) were fed with oak (and *Dioscorea* powder) pollen patty and the other colonies (*n* = 5) were fed with the rapeseed pollen patty. The pollen patties were crafted by a local farm using corresponding bee pollens. Bee pollen was sourced from the market and examined pollen morphology under microscope for verification. The pollen patties consisted of 70% bee pollen and 30% sugar solution, with the sugar solution concentration being 50% *w*/*w*. All other management practices were the same as common royal jelly production. No pollen traps were installed during the production, so that natural inflow of pollen was not restricted, as Korean royal jelly production is mostly executed in summer. During summer, wildflower blooming is limited, thus the influence of natural pollen inflow would be limited. Continuous monitoring of the apiary facilities was consistently maintained throughout the entire study duration. Proximate nutrient content of oak and rapeseed bee pollen is represented in Supplementary Table S1. Total protein contents of oak and rapeseed bee pollens were 23.2 and 26.8%, and total fat was 7.0 and 12.2%, respectively [31,32]. Royal jelly was produced following the standard procedure and collected from five beehives in each treatment group, storing the samples in glass bottles at a temperature of −20 °C. From storage, we sampled 5 random royal jelly bottles from each group for analyses.

### 2.2. Chemical Analyses

#### 2.2.1. Proximate and Amino Acid Analysis

All proximate nutrient contents, including moisture, crude protein, crude lipid, fiber, ash, and nitrogen free extract (NFE) of the RJ samples were estimated following standard methods of AOAC [31]. Moisture was estimated on an ‘as is’ basis, while the other proximate components were estimated on a dry weight basis. Royal jelly samples were dried using Christ Alpha 1-4LD Plus Freeze Dryer (CHRIST, Osterode am Harz, Germany). The amino acid composition was determined on an ‘as-is’ basis using a Sykam Amino Acid analyzer S433 (Sykam GmbH, Eresing, Germany) coupled with a Sykam LCA L-07 column, following the standard AOAC method [33]. RJ samples underwent hydrolysis in 6 N HCl for 24 h at 110 °C under a nitrogen atmosphere. Subsequently, the hydrolyzed samples were concentrated using a rotary evaporator, reconstituted with the manufacturer-supplied sample dilution buffer (physiological buffer 0.12 N citrate buffer, pH 2.20) and then introduced into the analyzer for amino acid anlaysis.

#### 2.2.2. 2D PAGE for Protein and MALDI-TOF

2D PAGE and protein identification by MALDI-TOF were conducted by the commercial facility of Genomine Inc, Pohang, South Korea. RJ samples were homogenized using a PowerGen125 homogenizer (Fisher Scientific, Waltham, MA, USA) with sample lysis solution [7 M urea, 2 M Thiourea containing 4% (*w*/*v*) 3-[(3-cholamidopropy)dimethyammonio]-1-propanesulfonate(CHAPS), 1% (*w*/*v*) dithiothreitol (DTT) and 2% (*v*/*v*) pharmalyte and 1 mM benzamidine]. After vortexing for one hour at room temperature and centrifugation, the soluble fraction was used for 2D gel electrophoresis. Protein concentration was determined by the Bradford method [34].

IPG dry strips (4–10 NL IPG, 24 cm, Genomine, Pohang, Republic of Korea) were equilibrated and loaded with the sample. Isoelectric focusing (IEF) was conducted using the Multiphor II electrophoresis unit and EPS 3500 XL power supply (Amersham Biosciences, Buckinghamshire, UK). After IEF, strips were incubated and inserted onto SDS-PAGE gels. SDS-PAGE was performed using the Hoefer DALT 2D system (Amersham Biosciences, Buckinghamshire, UK), and gels were stained using Colloidal CBB without glutaraldehyde following the method described by Oakley et al. [35].

Quantitative analysis using PDQuest software (version 7.0, BioRad, Hercules, CA, USA) normalized spot intensity, and protein spots with over two-fold expression variation compared to the control were selected. MALDI-TOF MS was employed for peptide identification following standard procedures [36].

#### 2.2.3. Fatty Acid and 10-HDA Analysis

The fatty acid composition on an ‘as-is’ basis was assessed using gas chromatography–flame ionization detection (GC-14B, Shimadzu, Tokyo, Japan) with an SP-2560 column, following the prescribed method in the Korean Food Standard Codex [37]. Samples were converted into fatty acid methyl esters (FAMEs). Identification and quantification of FAMEs were carried out by comparing retention times of peaks and peak areas with those of pure standards obtained from Sigma (Yongin, Republic of Korea) and analyzed under identical conditions. The determination of Trans-10-Hydroxy-2-Decenoic acid content of RJ samples followed the established procedure by Zhou et al. [38].

#### 2.2.4. Mineral Analysis

Mineral analysis on an ‘as is’ basis was conducted in adherence to established protocols outlined in the Korean Food Standard Codex [37]. The samples underwent digestion with a mixture of nitric and hydrochloric acid (1:3) at 200 °C for 30 min. The filtrate was stored in cleaned glass vials before subsequent analysis. Mineral content determination was performed utilizing an inductively coupled plasma optical emission spectrophotometer (ICP-OES 720 series; Agilent; Santa Clara, CA, USA).

### 2.3. Statistics

We followed the composite sampling procedure to collect samples for chemical analyses. In order to compare among the variables, we carried out an ANOVA and *t*-test where necessary using SPSS16.0. We reject the null hypothesis for *p* < 0.05 (CI = 95%).

## 3. Results

### 3.1. Proximate Composition

**Figure 1:**
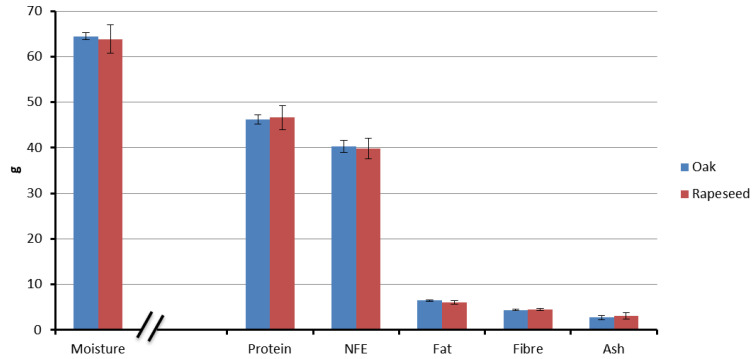
Comparison of the proximate component analyses of royal jelly samples derived from hives fed with oak and rapeseed pollen patty as feed supplements, respectively. Moisture was calculated on an ‘as is’ basis (g/100 g ‘as is’ basis), and other components were calculated on a dry weight basis (g/100 dry matter basis).

A comparative analysis of the proximate content of the royal jelly (RJ) samples is depicted in Figure 1. The moisture content of the RJ samples ranged from 59.4% to 68.2% (Supplementary Table S2). On a dry matter basis, the protein content fell within the range of 43.6 to 50.3%, while the lipid content of the RJ samples was notably low, accounting for 5.7 to 6.7%. Additionally, the fiber content ranged from 4.2 to 4.90%, and the ash content was reported to be between 1.9 and 3.7% (Supplementary Table S2).

Significantly, no notable differences in the proximate chemical composition were observed, except for fat, among the RJ samples obtained from the colonies that were fed on pollens from two distinct floral sources, namely oak and rapeseed (moisture: df = 8, t = 0.456, *p* = 0.330; protein: df = 8, t = −0.325, *p* = 0.376; fat: df = 8, t = 2.458, *p* = 0.019; fiber: df = 8, t = −0.86, *p* = 0.207; ash: df = 8, t = −0.931, *p* = 0.190) (Figure 1). 

### 3.2. Amino Acid Composition and Peptide

**Figure 2:**
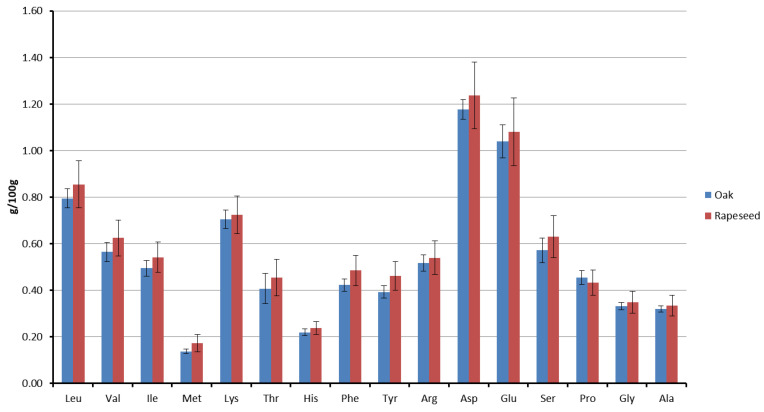
Amino acid composition (g/100 g ‘as is’ basis) of royal jelly samples derived from hives fed with oak and rapeseed pollen patties as feed supplements, respectively.

Figure 2 illustrates a comparative analysis of the amino acid content in the two distinct types of royal jelly (RJ) samples. Tryptophan and cysteine were not determined, likely due to the acid digestion method employed. The total amino acid content ranged from 7.93 to 10.46 g/100 g RJ (Supplementary Table S3). No significant differences were observed in both the individual (Figure 2) and total amino acid content of the RJ samples obtained from the colonies fed on pollens from two different floral sources (t = −0.976, df = 6, *p* = 0.367). All the essential amino acids, with the exception of tryptophan, were present in the RJ samples. Among the essential amino acids, leucine was the most abundant, followed by lysine. As for the non-essential amino acids, aspartic acid predominated, followed by glutamic acid.

The 2D electrophoresis analysis identified a total of 69 spots based on the molecular weight and isoelectric point, and Supplementary Table S4 provides a list of the separated proteins. Variations in the presence of proteins were observed between the two types of RJ, although no specific trend was established. Upon conducting the MALDI-TOF analysis based on the spot intensity, the MRJP1, MRJP2, MRJP3, and MRJP5 precursors and proteins were identified. Interestingly, the MRJP3 precursor and MRJP3 like protein densities were significantly higher in the oak treatment compared to the rapeseed treatment, with O/R (Oak/Rapeseed) ratios of 11 and 6.2, respectively (Table 1). Additionally, glucose oxidase was identified, showing a higher intensity in the oak RJ compared to the rapeseed RJ.

### 3.3. Fatty Acid Composition of 10-HDA Content

**Figure 3:**
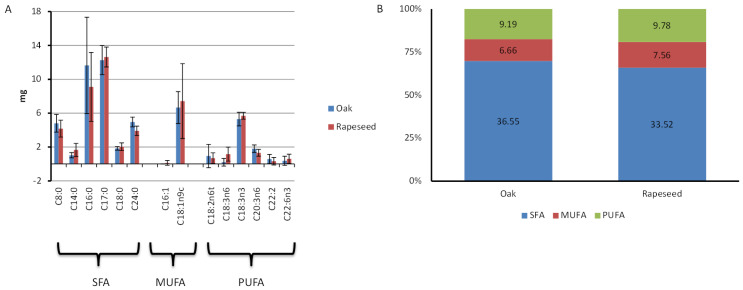
(**A**). Composition of saturated, monounsaturated, and polyunsaturated fatty acids (mg/100 g ‘as is’ basis) in royal jelly samples derived from hives fed with oak and rapeseed pollen patties as feed supplements, respectively. (**B**). Comparison of the saturated, monounsaturated, and polyunsaturated fatty acids (in %) of royal jelly samples derived from hives fed with oak and rapeseed pollen patties as feed supplements, respectively.

The fatty acid compositions of the two distinct types of royal jelly (RJ) samples are comparatively illustrated in Figure 3. The total content of fatty acids ranged from 44.27 to 66.33 mg/100 g of RJ (Supplementary Table S5). Similar to amino acids, there were no significant differences observed in either the total fatty acid contents between the two types of RJ (t = 0.291, df = 8, *p* = 0.779) or in any category of fatty acids (Figure 3). In most cases, saturated fatty acids (SFAs) were found to be abundant, followed by polyunsaturated fatty acids (PUFAs) and monounsaturated fatty acids (MUFAs), except in rapeseed royal jelly 4, where MUFAs were higher than PUFAs. Certain fatty acids, such as palmitoleic acid, docosadienoic acid, and docosahexaenoic acid were not consistently present in the RJ samples. Linoleic acid (cis) was absent in all the samples; however, linoelaidic acid (trans) was present, albeit inconsistently, in some RJ samples (Supplementary Table S5).

**Figure 4:**
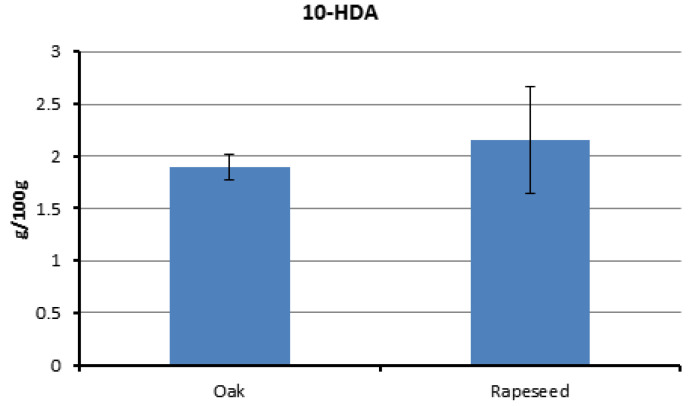
Comparative account of 10-HDA content (g/100 g ‘as is’ basis) of two different types of RJ samples derived from hives fed with oak and rapeseed pollen patties as feed supplements, respectively.

The content of 10-hydroxy-2-decenoic acid (10-HDA) is presented in Figure 4, with values ranging from 1.67 to 2.76 g/100 g of RJ (Supplementary Table S6). No statistically significant difference in the 10-HDA content was observed between the two types of RJ (rapeseed: 2.1 ± 0.51, oak: 1.9 ± 0.12 g/100 g; t = −1.057, df = 8, *p* = 0.321).

### 3.4. Mineral Content

**Figure 5:**
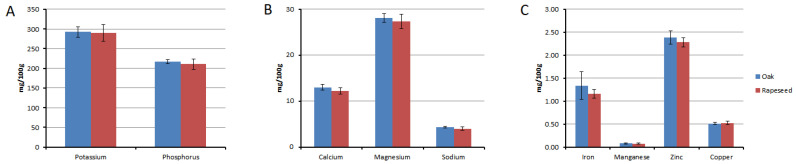
(**A**–**C**). Comparative account of mineral content (mg/100 g ‘as is’ basis) of two different types of RJ samples derived from hives fed with oak and rapeseed pollen patties as feed supplements, respectively.

Figure 5 illustrates the mineral content of the royal jelly (RJ) samples (Supplementary Table S7). No significant differences were observed between the two types of RJ for various minerals (calcium: t = 1.945, *p* = 0.088; magnesium: t = 0.877, *p* = 0.409; sodium: t = 1.489, *p* = 0.187; potassium: t = 0.265, *p* = 0.799; phosphorus: t = 0.981, *p* = 0.371; iron: t = 1.231, *p* = 0.273; manganese: t = 0.849, *p* = 0.424; zinc: t = 1.296, *p* = 0.236; copper: t = −0.728, *p* = 0.494). The most abundant minerals were potassium (rapeseed: 289.6 ± 20.80, oak: 292.5 ± 13.22 mg/100 g) and phosphorus (rapeseed: 210.4 ± 13.46, oak: 216.7 ± 4.94 mg/100 g), followed by magnesium (rapeseed: 27.4 ± 1.53, oak: 28.1 ± 0.91 mg/100 g). Among the macro minerals, the sodium content was the lowest (rapeseed: 4.0 ± 0.38, oak: 4.3 ± 0.19 mg/100 g). Among the micro minerals, zinc predominated (rapeseed: 2.3 ± 0.10, oak: 2.4 ± 0.15 mg/100 g), followed by iron (rapeseed: 1.2 ± 0.09, oak: 1.3 ± 0.31 mg/100 g), copper (rapeseed: 0.5 ± 0.04, oak: 0.5 ± 0.02 mg/100 g), and manganese (0.1 ± 0.01 mg/100 g for rapeseed and oak).

## 4. Discussion

Honey bee colonies were provided with feeds of varying nutritional value: oak and rapeseed pollen patties. Despite differences in the nutrient composition between the two bee pollens and their respective patties, there were no statistical significant variations, except for the crude fat, in the nutritional content of the resulting royal jelly (RJ) from the colonies fed with different provisions (Figure 1, Figure 2, Figure 3, Figure 4 and Figure 5). Despite the rapeseed pollen exhibiting a higher fat content compared to the oak pollen [31,32], this discrepancy was not reflected in the respective royal jelly. The consistent nutrient content in both types of royal jelly suggests an adaptive capacity to meet nutritional standards, ensuring the dietary needs of the brood and queen during bee pollen collection, preserving both bee bread and royal jelly secretion. The absence of restrictions on incoming bee pollen might allow for potential blending with the feed. However, during the summer, wildflower bloomings are limited, thus restricting the extent of its influence. A previous study noted that a pollen patty made from bee pollen contained lower protein and fat content than the corresponding bee pollen [32]. Additionally, when honey bees transform the feed (in this case, pollen patty) into bee bread, the nutritional composition undergoes a transformation [39]. The collection and storage process of pollen by honey bees also alters the nutritional composition of pollen [40]. Hence, the alteration of the feed could serve as a plausible explanation for the production of royal jelly with a similar quality. The process of transforming bee pollen or pollen patties into bee bread might involve a compensation mechanism in honey bees to maintain the nutritional content. Balancing feed with adequate nutrients is crucial to prevent negative consequences on honey bee physiology. Our investigation reveals that, within certain levels, honey bees can compensate for differences in the nutrient content between different bee feeds and therefore, no differences in nutritional quality were observed between two different types of royal jelly.

The obtained values for the moisture content, falling within the range of 59.4 to 68.2%, align with those reported in previous studies (51.03 to 69.7%), with variations attributed to the age of the royal jelly and the season of collection [41,42]. Protein constitutes approximately 50% of royal jelly (RJ) based on the dry weight [43], which we found to be true in the present study. Protein, comprising proteinogenic amino acids, serves various vital roles in animal physiology. The protein levels in the oak and rapeseed bee pollen were 23.2% and 26.8%, respectively. Nevertheless, when the bees were fed on pollen patties containing these two types of bee pollens, the resulting royal jelly (RJ) samples from two distinct hives did not exhibit significantly different amino acid contents. Essential amino acids, obtained from the diet, are particularly crucial during early life, such as the growth period. In contrast, adults primarily require amino acids for fundamental somatic functions and/or reproduction, and their demand for essential amino acids diminishes with age [44,45]. Unlike adults, larvae have a higher protein and amino acid requirement. However, the protein content in the diet for young larvae fluctuates with their age and is subject to seasonal variations [46]. Considering the total amino acids as the protein content, the obtained results (ranging from 7.9 to 10.5 g/100 g RJ) align with published reports on the protein content (9 to 18%) of fresh royal jelly (RJ) [7]. The proportion of essential amino acids (54.4% for oak RJ and 55.6% for rapeseed RJ) was found to be higher than that of non-essential amino acids in both types of RJ. The distribution of amino acids in the RJ concurred with previous reports, where aspartic acid was identified as the most abundant, followed by glutamic acid and leucine [47].

The primary component of this protein is major royal jelly protein (MRJP), which comprises nine different types, with MRJP1–3 and 5 being the most abundant [29], as corroborated by our study. Two-dimensional electrophoresis revealed varying concentrations (based on protein spot intensity) for the RJ samples obtained from the hives supplemented with oak and rapeseed pollen patties. Interestingly, in some cases, the protein intensity was significantly higher in the RJ obtained from the hives supplemented with oak pollen patties compared to those supplemented with rapeseed pollen patties, which contradicts the protein content of these two pollens. The observed variation, represented by the ratio of the spot intensity of protein in the two different types of RJ samples, may be attributed to the influx of bee pollen. However, further investigations are required to determine the extent of this impact. MRJPs play a crucial nutritional role in the diet of queen honey bees. Specifically, MRJP1, MRJP4, and MRJP5 serve as the primary source of essential amino acids [5,48,49,50,51], while MRJP2 and MRJP5 act as significant nitrogen reserves for larval growth. MRJP3, exhibiting size polymorphism, contributes to the nitrogen supply [49,52]. Moreover, MRJP1 and MRJP2 exhibit antibacterial activity [53,54] and have been identified as major allergens in RJ, stimulating the production of TNF-α in mouse macrophages in vivo [55,56].

Pollen consumption holds significant importance in this context, serving as the primary source of protein, fat, and minerals for honey bees. Previous studies have indicated that the efficient production of royal jelly protein relies on the basic upregulation of MRJP expression and increased acini volumes, enabling higher protein production in nurse bee hypopharyngeal glands [29,57]. Pollen consumption enhances vitellogenin expression and increases vitellogenin levels in hemolymph [58]. The upregulation of MRJP is likely dependent on vitellogenin. Additionally, the consumption of pollen leads to an increase in the size of the hypopharyngeal glands [26]. Therefore, the protein content of pollen plays a crucial role in the context of royal jelly production.

10-HDA stands out as the primary fatty acid compound in royal jelly (RJ) and constitutes the most pivotal component of RJ. In the current study, the obtained 10-HDA values in the RJ samples were either similar or slightly lower than those reported by Wang et al. [42]. Notably, all the RJ samples contained 10-HDA levels surpassing the suggested quality standardization for RJ [7]. Wang et al. [42] highlighted a decline in the amount of 10-HDA with increasing storage days, and another study demonstrated a 0.4% and 0.6% reduction in 10-HDA in two RJ samples stored at room temperature for 12 months [59]. Beyond serving as a marker component, 10-HDA plays a crucial role in various biological activities associated with colony development strategies. Crailsheim and Riessberger-Gallé [60] showed that different concentrations of RJ and worker jelly inhibit the growth of *Paenibacillus larvae*. Additionally, it has been suggested that 10-HDA exhibits antipathogenic activity in larval food within the midgut of young larvae, contributing to larval resistance against *P. larvae* [61]. However, the inhibitory activity of 10-HDA is influenced by the pH [61]. The persistent occurrence of n-3 PUFA, particularly linolenic acid, underscores its significance, as evidenced by prior research showing that bees subjected to linolenic-acid-deficient diets exhibit a reduced hypopharyngeal gland size and impaired learning acquisition [62], while the intake of diets with high ω6:ω3 ratios leads to elevated mortality and diminished brood rearing capacity [63].

The obtained values for the ash content of the royal jelly (RJ) fall within the range reported in previous studies [7,64]. Consistent with our present findings, potassium emerged as the most abundant mineral in the RJ, aligning with other published reports [11,42,65]. Notably, our study revealed an exceptionally low amount of sodium. The presence of minerals is primarily influenced by external factors such as food sources, pollen quality, production periods, and environmental conditions [11].

## 5. Conclusions

Royal jelly, a remarkable substance produced by nurse honey bees, plays a crucial role within the hive, particularly in the development and sustenance of the queen bee, who, as the hive’s reproductive center, is vital for the colony’s survival and growth. Royal jelly is fed to her throughout her entire life, endowing her with enhanced size, longevity, and fertility compared to worker bees. Ensuring the hive’s prosperity relies on the honey bee’s ability to compensate for variations in input quality. One possible mechanism of compensation involves the production of nutritionally balanced foods, such as bee bread. Nurse bees feeding on the bee bread produce royal jelly that is consistent with its nutritional quality. However, the full extent of this compensation is yet to be determined, as the quality of the oak and rapeseed pollen did not differ highly and both of them are often used as bee feed ingredients. An alternative possibility could be that royal jelly results from the amalgamation of secretions from the hypopharyngeal and mandibular glands, suggesting that there is no immediate direct conversion of pollen into royal jelly. Assessing the impact on the quality of royal jelly when the feed quality varies significantly remains a task for further investigation.

## Figures and Tables

**Table 1:** Protein separation, spot intensity (mean ± SD), and selected identification of protein of royal jelly samples by MALDI-TOF showing O/R > 2.

Standard Spot	Molecular Weight	Isoelectric Point	Spot Intensity		Protein Identified	O/R *
			Oak	Rapeseed		
2001	17.50	6.25	55.2 ± 39.5	26.2 ± 23.2		2.1
2201	45.40	5.89	474.4 ± 480.9	75.6 ± 166.8		6.3
4606	78.04	6.91	3971.4 ± 5532.4	1519.0 ± 1097.8		2.6
6101	29.17	7.21	461.9 ± 297.8	127.8 ± 231.8		3.6
6802	109.36	7.28	50.7 ± 54.0	17.3 ± 36.5		2.9
7101	29.34	7.54	461.9 ± 297.8	41.8 ± 39.6	MRJP3 precursor	11.0
7503	68.26	7.84	1084.4 ± 591.0	416.3 ± 280.9	MRJP3	2.6
7701	87.58	7.48	341.1 ± 251.9	131.1 ± 105.0	Glucose oxidase	2.6
7702	79.52	7.63	1704.3 ± 877.1	843.5 ± 451.1		2.0
8103	34.82	8.13	301.0 ± 389.0	45.3 ± 27.2		6.6
8106	44.39	8.26	470.0 ± 415.1	76.0 ± 42.0	MRJP3 like	6.2
8801	80.69	7.91	107.6 ± 98.7	34.0 ± 73.7		3.2
8803	88.11	7.67	30.9 ± 34.1	14.0 ± 17.9		2.2

* O/R = oak/rapeseed.

## Data Availability

We have supplied all the data in the Supplementary Materials.

## Supplementary Materials

The following supporting information can be downloaded at https://www.mdpi.com/article/10.3390/insects15030141/s1, Table S1: Nutrient composition of oak and rapeseed pollen (obtained from Ghosh and Jung 2017, 2020 [31,32]); Table S2: Moisture (fresh weight basis) and proximate component analyses (% dry matter basis) of royal jelly samples; Table S3: Amino acid composition (g/100 g as is basis, mean ± SD) of royal jelly samples; Table S4: Protein separation, spot intensity (mean ± SD), and selected identification of protein of royal jelly samples by MALDI-TOF; Table S5: Fatty acid composition (mg/100 g as is basis) of royal jelly samples; Table S6: 10-HDA content (g/100 g as is basis) of royal jelly samples; Table S7: Minerals content (mg/100 g as is) of royal jelly samples
