# Supplementary material for: Chemical Composition and Nutritional Value of Royal Jelly Samples Obtained from Honey Bee (Apis mellifera) Hives Fed on Oak and Rapeseed Pollen Patties
Source: Insects. 2024 Feb 21;15(3):141. doi: 10.3390/insects15030141 (PMC10970897; doi:10.3390/insects15030141)
Supplement: Supplementary file 1 [file insects-15-00141-s001.zip › insects-2842302-supplementary.pdf]

Table S1: Nutrient composition of oak and rapeseed pollen (obtained from Ghosh and Jung 2017, 2020 [31,32])

	Oak pollen	Rapeseed pollen
**Moisture**	4.4 ± 0.19	4.54
**Protein**	23.2 ± 0.10	26.8± 0.07
**Fat**	7.0 ± 0.19	12.2±0.04
**Fibre**	4.2 ± 0.53	6.2±0.08
**Ash**	5.3 ± 0.13	5.3±0.03
**NFE**	60.2 ± 0.92	49.5±0.03

Table S2: Moisture (fresh weight basis) and proximate component analyses (% dry matter basis) of royal jelly samples

	**Oak RJ**					**Rapeseed RJ**				
	**1**	**2**	**3**	**4**	**5**	**1**	**2**	**3**	**4**	**5**
Moisture	64.00	64.37	65.36	65.05	63.54	63.89	59.42	63.15	68.17	64.41
Protein	46.4±0.72	44.9±0.23	45.6±0.77	46.5±1.14	47.5±0.32	50.3±2.78	44.7±0.15	46.1±0.67	48.3±1.14	43.6±0.39
NFE	40.7±1.07	41.5±0.35	41.1±0.59	39.8±0.56	38.4±0.50	36.4±2.88	41.3±0.23	40.1±0.24	39.1±1.53	42.2±0.04
Fat	6.5±0.11	6.3±0.06	6.3±0.05	6.5±0.17	6.7±0.06	6.5±0.31	5.7±0.03	5.8±0.11	6.3±0.10	5.9±0.12
Fibre	4.5±0.04	4.6±0.03	4.4±0.10	4.3±0.04	4.2±0.02	4.3±0.20	4.7±0.06	4.4±0.03	4.3±0.02	4.9±0.01
Ash	1.9±0.43	2.8±0.05	2.5±0.13	3.0±0.31	3.3±0.29	2.6±0.20	3.7±0.36	3.5±0.57	2.1±0.38	3.4±0.55

Table S3: Amino acid composition (g/100g as is basis, mean ± SD) of royal jelly samples

	**Oak RJ**				**Rapeseed RJ**			
	**1**	**2**	**3**	**4**	**1**	**2**	**3**	**4**

Leucine*	0.79	0.74	0.82	0.83	0.91	0.76	0.97	0.78
Valine*	0.61	0.51	0.57	0.57	0.67	0.55	0.71	0.57
Isoleucine*	0.49	0.45	0.51	0.53	0.57	0.48	0.62	0.50
Methionine*	0.15	0.14	0.13	0.13	0.17	0.13	0.22	0.17
Lysine*	0.72	0.66	0.75	0.69	0.80	0.66	0.79	0.65
Threonine*	0.43	0.35	0.49	0.36	0.47	0.36	0.55	0.44
Histidine*	0.22	0.20	0.23	0.23	0.25	0.21	0.27	0.22
Phenylalanine*	0.40	0.40	0.44	0.45	0.50	0.42	0.57	0.45
Tyrosine**	0.43	0.39	0.37	0.38	0.49	0.39	0.53	0.44
Arginine	0.52	0.47	0.55	0.53	0.58	0.47	0.62	0.49
Aspartic acid	1.18	1.12	1.19	1.22	1.34	1.10	1.38	1.13
Glutamic acid	1.02	0.95	1.08	1.11	1.18	0.94	1.23	0.97
Serine	0.57	0.51	0.64	0.57	0.67	0.52	0.73	0.60
Proline	0.42	0.44	0.48	0.48	0.45	0.36	0.49	0.43
Glycine	0.34	0.31	0.34	0.34	0.38	0.31	0.40	0.31
Alanine	0.33	0.30	0.32	0.33	0.35	0.29	0.39	0.31
**Total**	**8.63**	**7.93**	**8.91**	**8.75**	**9.78**	**7.94**	**10.46**	**8.46**

Cysteine and tryptophan were not determined

*Essential amino acid; **Conditional essential amino acid

Table S4: Protein separation, spot intensity (mean ± SD) and selected identification of protein of royal jelly samples by MALDI-TOF.

Standard spot	Molecular weight	Isoelectric point	Spot intensity		Protein identified	O/R*
			Oak	Rapeseed		
406	48.41	4.70	476.0±216.6	542.9±41.0		0.9

801	75.20	4.85	1190.7±589.8	1131.8±590.7		1.1
1004	22.29	5.80	16419.6±1747.6	13720.9±4143.7	MRJP1	1.2
1303	56.15	5.11	5775.0±132.2	4915.4±1333.9		1.2
1306	50.97	4.99	698.0±1.2	596.3±221.4		1.2
1405	59.28	5.20	207.4±76.6	181.5±114.0		1.1
1602	55.55	5.00	27438.3±4251.6	23766.2±10429.0	MRJP1 precursor	1.2
1705	66.63	5.04	682.0±183.0	639.7±164.6		1.1
1801	73.54	4.98	456.9±153.0	445.0±205.3		1.0
1802	74.34	4.92	597.2±315.8	744.1±392.8		0.8
2001	17.50	6.25	55.2±39.5	26.2±23.2		2.1
2102	44.23	6.26	2539.3±993.9	2081.9±1032.9		1.2
2103	40.31	6.26	143.4±82.1	88.8±69.7		1.6
2201	45.40	5.89	474.4±480.9	75.6±166.8		6.3
2202	50.75	5.95	951.2±99.6	873.0±267.4		1.1
2203	50.47	6.22	858.6±280.0	834.6±351.9		1.0
2204	45.35	5.97	1414.4±66.1	1083.3±531.4		1.3
2503	61.54	6.14	4115.4±1237.3	4132.9±1001.2		1.0
2602	77.39	6.14	6297.4±2466.6	7918.9±1066.8	MRJP5 precursor	0.8
3002	22.48	6.46	741.2±321.4	790.0±110.9		0.9
3003	16.24	6.40	331.3±107.5	395.4±35.5		0.8
3201	50.13	6.50	404.3±246.5	324.1±206.8		1.2
3302	49.18	6.34	157.7±87.6	180.7±8.3		0.9
3601	76.86	6.39	4774.6±1388.6	5825.8±998.2		0.8
4201	44.75	6.60	247.3±187.6	311.8±123.7		0.8
4401	44.79	6.46	80.7±89.8	80.9±45.2		1.0
4502	68.41	6.79	699.0±410.0	484.1±480.9		1.4

4601	77.65	6.65	2749.4±1192.7	3238.6±1546.1		0.8
4604	78.35	6.82	691.3±428.6	1703.0±1186.4		0.4
4606	78.04	6.91	3971.4±5532.4	1519.0±1097.8		2.6
5001	15.19	6.96	110.4±52.4	83.2±35.1		1.3
5102	35.74	7.11	257.1±53.7	313.3±166.2		0.8
5103	33.66	7.09	255.6±73.5	219.3±127.8		1.2
5202	39.88	6.95	170.1±160.2	185.4±104.4		0.9
5401	46.68	6.97	482.4±479.2	728.1±3.2		0.7
5502	68.44	7.02	1066.4±588.4	673.7±656.1		1.6
5503	65.16	7.07	5513.0±1739.5	5116.2±2477.7	MRJP3 precursor	1.1
5601	78.40	7.08	1345.9±42.1	2081.8±1135.8		0.6
5603	78.58	7.16	1619.8±546.9	1419.9±826.3		1.1
5604	61.39	6.72	705.8±382.3	755.6±213.3		0.9
6101	29.17	7.21	461.9±297.8	127.8±231.8		3.6
6202	39.91	7.23	144.0±116.6	122.2±119.4		1.2
6501	67.97	7.21	1066.4±588.4	673.7±656.1		1.6
6502	64.99	7.29	7153.0±2677.8	5128.2±2526.8		1.4
6503	68.06	7.41	1066.8±583.9	674.3±656.3		1.6
6703	78.89	7.35	2076.5±619.9	1484.4±688.5		1.4
6801	110.22	7.15	50.5±50.2	27.2±58.7		1.9
6802	109.36	7.28	50.7±54.0	17.3±36.5		2.9
7001	13.46	7.40	59.3±47.3	35.0±23.3		1.7
7101	29.34	7.54	461.9±297.8	41.8±39.6	MRJP3 precursor	11.0
7203	40.22	7.48	114.5±67.8	66.4±89.7		1.7
7401	64.77	7.74	7235.4±2749.0	5236.2±2521.6		1.4
7501	64.99	7.51	7273.0±2781.8	5168.2±2604.9		1.4

7502	68.18	7.63	1087.2±589.1	714.3±618.9		1.5
7503	68.26	7.84	1084.4±591.0	416.3±280.9	MRJP3	2.6
7701	87.58	7.48	341.1±251.9	131.1±105.0	Glucose oxidase	2.6
7702	79.52	7.63	1704.3±877.1	843.5±451.1		2.0
8101	38.83	8.25	1709.5±909.9	1311.0±764.9		1.3
8102	36.86	8.16	619.8±334.9	787.1±549.2		0.8
8103	34.82	8.13	301.0±389.0	45.3±27.2		6.6
8106	44.39	8.26	470.0±415.1	76.0±42.0	MRJP3 like	6.2
8107	41.39	8.25	18.0±38.1	20.7±44.0		0.9
8302	52.77	8.10	6705.4±2119.9	4441.9±1838.1	MRJP2 precursor	1.5
8401	64.51	7.97	7275.4±2783.9	5242.2±2514.5		1.4
8502	66.43	8.19	5754.8±3132.2	3941.7±2885.3		1.5
8801	80.69	7.91	107.6±98.7	34.0±73.7		3.2
8803	88.11	7.67	30.9±34.1	14.0±17.9		2.2
9601	73.77	8.65	4681.1±2605.4	3671.3±1361.4		1.3
9604	58.38	8.45	62.4±35.4	41.3±61.5		1.5

*O/R = Oak/Rapeseed

Table S5: Fatty acid composition (mg/100g as is basis) of royal jelly samples

Fatty acids	Oak					Rapeseed				
	1	2	3	4	5	1	2	3	4	5
Caprylic acid (C8:0)	3.78	4.92	6.43	4.03	4.87	3.69	3.93	5.45	4.87	2.92
Myristic acid (C14:0)	0.69	1.16	1.49	0.89	0.93	3.03	1.32	1.11	1.36	1.49
Palmitic acid (C16:0)	19.30	9.77	9.64	4.45	15.04	7.87	8.21	5.73	16.18	7.51

Heptadecanoic acid (C17:0)	10.77	11.64	15.18	12.30	11.45	12.16	11.63	14.54	12.95	11.90
Stearic acid (C18:0)	1.94	1.72	1.86	1.56	2.13	1.76	2.12	1.96	2.76	1.58
Lignoceric acid (C24:0)	5.71	5.23	5.08	4.26	4.53	4.04	3.46	4.81	3.47	3.79
**Subtotal (SFA)**	**42.19**	**34.44**	**39.68**	**27.49**	**38.95**	**32.55**	**30.67**	**33.60**	**41.59**	**29.19**
Palmitoleic acid (C16:1)	0.00	0.00	0.00	0.00	0.00	0.00	0.00	0.00	0.64	0.00
Oleic acid (C18:1n9c)	7.74	6.16	5.68	4.44	9.28	5.99	7.87	3.34	14.79	5.15
**Subtotal (MUFA)**	**7.74**	**6.16**	**5.68**	**4.44**	**9.28**	**5.99**	**7.87**	**3.34**	**15.43**	**5.15**
Linolelaidic acid (C18:2n6t)	3.03	1.66	0.00	0.00	0.00	1.30	0.00	0.95	0.00	1.16
Linoleic acid (C18:2n6c)	0.00	0.00	0.00	0.00	0.00	0.00	0.00	0.00	0.00	0.00
r-Linolenic acid (C18:3n6)	0.00	1.00	0.00	0.00	0.00	2.19	0.00	0.85	1.59	1.17
Linolenic acid (C18:3n3)	6.05	4.52	5.80	5.80	4.34	5.61	6.22	5.34	5.31	5.99
cis-11,14-Eicosadienoic acid (C20:2)	0.00	0.00	0.00	0.00	0.00	0.00	0.00	0.00	0.00	0.00
cis-8,11,14-Eicosatrienoic acid (C20:3n6)	1.53	2.24	2.34	1.47	1.42	1.49	0.94	1.86	1.37	0.92
cis-13,16-Docosadienoic acid (C22:2)	1.12	0.00	0.97	0.00	0.81	0.00	0.00	0.91	0.00	0.69
cis-4,7,10,13,16,19-Docosahexaenoic acid (C22:6n3)	0.00	0.00	0.00	1.17	0.70	1.10	0.00	0.88	1.04	0.00
**Subtotal (PUFA)**	**11.73**	**9.42**	**9.11**	**8.44**	**7.27**	**11.69**	**7.16**	**10.79**	**9.31**	**9.93**
**Total**	**61.66**	**50.02**	**54.47**	**40.37**	**55.50**	**50.23**	**45.70**	**47.73**	**66.33**	**44.27**

Table S6: 10-HDA content (g/100g as is basis) of royal jelly samples

		10HDA
Rapeseed	1	1.74
	2	1.67
	3	2.61
	4	2.76
	5	1.95

Oak	1	2.09
	2	1.83
	3	1.92
	4	1.76
	5	1.90

Table S7: Minerals content (mg/100g as is) of royal jelly samples

		Calcium	Magnesium	Sodium	Potassium	Phosphorus	Iron	Manganese	Zinc	Copper
	**1**	12.53	27.35	4.08	274.87	214.94	1.80	0.07	2.55	0.51
	**2**	13.36	28.59	4.30	283.53	218.47	1.48	0.10	2.50	0.54
**Oak**	**3**	13.56	28.17	4.39	299.35	213.47	1.18	0.09	2.25	0.49
	**4**	13.33	29.24	4.11	308.06	224.45	1.03	0.08	2.40	0.52
	**5**	12.18	27.00	4.54	296.83	212.14	1.20	0.07	2.22	0.51
	**1**	11.55	26.08	4.63	265.79	196.24	1.25	0.07	2.24	0.50
	**2**	11.83	26.66	3.81	285.28	208.00	1.19	0.08	2.26	0.50
**Rapeseed**	**3**	11.96	26.57	3.91	276.09	200.33	1.00	0.07	2.18	0.50
	**4**	12.33	27.61	3.63	303.62	218.14	1.19	0.07	2.29	0.58
	**5**	13.32	29.93	4.02	317.28	229.29	1.18	0.09	2.44	0.56
